# Different Functional Network Connectivity Patterns in Epilepsy: A Rest-State fMRI Study on Mesial Temporal Lobe Epilepsy and Benign Epilepsy With Centrotemporal Spike

**DOI:** 10.3389/fneur.2021.668856

**Published:** 2021-05-28

**Authors:** Cong Fu, Aikedan Aisikaer, Zhijuan Chen, Qing Yu, Jianzhong Yin, Weidong Yang

**Affiliations:** ^1^Department of Neurosurgery, Tianjin Medical University General Hospital, Tianjin, China; ^2^Department of Radiology, Tianjin First Central Hospital, Tianjin, China; ^3^Department of Neurology, Tianjin Medical University General Hospital, Tianjin, China

**Keywords:** resting state networks, mesial temporal lobe epilepsy, benign epilepsy with centrotemporal spikes, BOLD fMRI, functional network connectivity

## Abstract

The stark discrepancy in the prognosis of epilepsy is closely related to brain damage features and underlying mechanisms, which have not yet been unraveled. In this study, differences in the epileptic brain functional connectivity states were explored through a network-based connectivity analysis between intractable mesial temporal lobe epilepsy (MTLE) patients and benign epilepsy with centrotemporal spikes (BECT). Resting state fMRI imaging data were collected for 14 MTLE patients, 12 BECT patients and 16 healthy controls (HCs). Independent component analysis (ICA) was performed to identify the cortical functional networks. Subcortical nuclei of interest were extracted from the Harvard-Oxford probability atlas. Network-based statistics were used to detect functional connectivity (FC) alterations across intranetworks and internetworks, including the connectivity between cortical networks and subcortical nuclei. Compared with HCs, MTLE patients showed significant lower activity between the connectivity of cortical networks and subcortical nuclei (especially hippocampus) and lower internetwork FC involving the lateral temporal lobe; BECT patients showed normal cortical-subcortical FC with hyperconnectivity between cortical networks. Together, cortical-subcortical hypoconnectivity in MTLE suggested a low efficiency and collaborative network pattern, and this might be relevant to the final decompensatory state and the intractable prognosis. Conversely, cortical-subcortical region with normal connectivity remained well in global cooperativity, and compensatory internetwork hyperconnectivity caused by widespread cortical abnormal discharge, which might account for the self-limited clinical outcome in BECT. Based on the fMRI functional network study, different brain network patterns might provide a better explanation of mechanisms in different types of epilepsy.

## Introduction

Epilepsy derives from the long-term spontaneous abnormal discharge of neurons in the brain, resulting in hypersynchronization of the cortical-cortical and subcortical-cortical regions, thus leading to brain dysfunction and behavioral abnormalities. About 25% epilepsy patients with a dissatisfied clinical control of seizure even with the optimal anti-epileptic drugs (AEDs) (1). The most common drug-resistant epilepsy in adults is mesial temporal lobe epilepsy (MTLE) (2), accounting for 80% of temporal lobe onset seizures (3). Inversely, some of the epilepsy patients have a good response to AEDs and even achieve a seizure-free result, such as benign epilepsy with centrotemporal spikes (BECT). BECT is the most common form of childhood focal epilepsy and is usually idiopathic without structural brain abnormalities (4).

The drug-resistance epilepsy might relate to brain decompensatory processes (5) and self-limited epilepsy might contribute to compensatory cortical reorganization (6, 7). Therefore, patients with MTLE and BECT might mark different prognosis by different brain compensatory patterns. The two patterns manifest brain networks abnormalities usually caused by epileptic discharges in widespread brain areas in MTLE and BECT. Thus, resting state functional connectivity (RSFC) could be used to detect the network -level epileptic effect.

Routine EEG examination and different imaging methods drive the conclusion that epilepsy is a network disease that is not only confined to the epileptogenic zones but also involved in widespread cortical and subcortical disturbances (8, 9). Ictal EEG performance suggests that MTLE primarily involves the temporal lobes, and the abnormal network is known to have widespread extratemporal connectivity, such as the lateral temporal, insular, and frontal regions (2, 10, 11). Imaging observations have suggested the presence of one or more common subcortical sources of widespread network dysfunction in MTLE. Hippocampal sclerosis is very significant and the most common pathological feature of MTLE. Moreover, the thalamus directly connected to the hippocampus has been shown to suffer atrophy (12, 13). In addition, chronic network changes associated with MTLE have been identified by impaired RSFC within the hippocampus and enhanced RSFC within the medial temporal lobe with extensions to the lateral temporal lobes (10, 14).

In the same vein, patients with BECT were found to have bilateral frontal and parieto-occipital regions that showed spectral changes in a resting-state EEG study (15). A growing body of literature examining cognitive and behavioral outcomes by imaging methods suggests that BECTS children perform less well-than their peers (16), including worse attention and visuomotor performance (17, 18) and reversible speech and cognitive dysfunction (19, 20).

MTLE and BECT both suffer from neural abnormal discharges while their prognosis is obviously different. Moreover, alterations in the brain functional networks related to epileptic prognosis remain to be fully clarified, especially in the state of epileptic compensation. Thus, the fMRI approach was used to find the changes in functional networks and probably pathological mechanisms. We speculated that the network-based approach would be promising for revealing the complex network patterns to explain the mechanisms underlying the different prognosis in epilepsy.

## Materials and Methods

### Participants

Fourteen MTLE patients and twelve BECT patients were recruited from the Epilepsy Clinic of Neurology and Neurosurgery Departments in Tianjin Medical University General Hospital. The diagnoses of MTLE and BECT were established by history, clinical symptoms, magnetic resonance imaging (MRI), and video electroencephalogram (VEEG) by 2 senior epileptologists (Q.Y. and Z.C.). The inclusion criteria for patients with MTLE and BECT were as follows: (1) typical clinical manifestations and specific EEG characteristics according to International League Against Epilepsy (ILAE) (21); (2) the presence of routine clinical scans, including high-resolution 3D T1-weighted and FLAIR MRI and high in-plane resolution 2D coronal T2-weighted MRI according to the Harmonized Neuroimaging Of Epilepsy Structural Sequences (HARNESS) (22); (3) no evidence of other structural brain abnormalities due to hypoplasia of brain parenchyma, brain trauma, tumor, etc; and (4) MTLE patients should be diagnosed as the drugs resistance epilepsy (23) and the patients with BECT should respond well to AEDs. Patients in both groups received oxcarbazepine/carbamazepine for seizures treatment.

For the lesion lateralization, there were 2 on right MTL and 3 on left MTL, and the rest of our drug-resistant MTLE patients were failed to detect the epileptogenic focus. And all of the BECT patients were bilateral abnormal discharges in EEG and we could not find the stationary focus on one side. Patient demographics and a clinical summary are shown in Table 1. A healthy control group (*n* = 16) was matched by demographic characteristics from the local community. None of the HCs had a history of neurological or mental illness. The study was approved by the ethics committee of Tianjin Medical University General Hospital and completed according to the standards established in the Helsinki Declaration. Each subject gave written informed consent in accordance with the Hospital Research Ethics Committee.

**Table 1:** Demographic and clinical characteristics of all participants.

**Characteristics**	**Groups**			***p***
	**HCs *n* = 16**	**MTLE *n* = 14**	**BECT *n* = 12**	
Male: Female (*n*)	6:10	5:9	5:7	0.95
**Age (years)**				
Mean ± SD	27.1 ± 4.8	35.36 ± 17.2	10.42 ± 4.5	<0.001
**Epilepsy Duration**				
**(years)**				
Mean ± SD		17.28 ± 8.16	4.62 ± 4.1	<0.001
**Seizure type (*****n*****)**				
SPS: CPS: SGTCS		1:12:1	2:5:5	0.06
**Interictal EEG (*****n*****)**				
BCT: +CFT: +CPT		—	7:3:2	
Sph1: Sph2		7:7	—	

*HCs, healthy controls; MTLE, mesial temporal lobe epilepsy; BECT, benign epilepsy with centrotemporal spikes; SPS, simple partial seizure; CPS, complex partial seizure; SGTCS, secondary generalized tonic-clonic seizure; BCT, bilateral centro-temporal; +CPT, BCT and centro-parieto-temporal; +CFT, BCT and centro-fronto-temporal; Sph1, left sphenoid electrode; Sph2, right sphenoid electrode*.

### MRI Acquisition

All MRI scanning data were obtained on a 3-Tesla MRI scanner (Siemens Trio Tim). High-resolution T1-weighted data images were acquired using a magnetization-prepared rapid gradient echo (MPRAGE) sequence (repetition time (TR) = 1,900 ms, echo time (TE) = 2.52 ms, field of view (FOV) = 256 mm × 256 mm, matrix 256 × 256, slice thickness 1 mm, 176 volumes). Resting-state functional blood oxygen level-dependent (12) data images were acquired using an echo planar imaging sequence (TR = 2,000 ms, TE = 30 ms, flip angle 90°, FOV = 220 mm × 220 mm, matrix 80 × 80, slice thickness 5 mm, 300 volumes). The patients were asked to not move and to stay with eyes closed and resting. Headphones and cushions were used to reduce noise interference and prevent excessive head movement.

### Network-Based Functional MRI (fMRI) Analysis

#### Resting-State fMRI Preprocessing

Preprocessing of the data was performed according to the Graph-theoretical Network Analysis Toolkit (GRETNA) (http://www.nitrc.org/projects/gretna/) fMRI preprocessing pipeline. The first 10 volumes were removed, and then slice-timing correction and head motion correction were performed. The data from patients with head motion exceeding 2 mm or head rotations <2° were excluded from further calculations, while head motion in controls was limited 1 mm or 1°,The motion-corrected functional images were normalized to the standard Montreal Neurological Institute (MNI) space by applying an EPI template at a 3 × 3 × 3 mm^3^ resolution, which led to our data showing a better match with the EPI template (24). Subsequently, to avoid mixing white matter and gray matter signals, the normalized images were spatially smoothed using a 4-mm full-width half-maximum Gaussian kernel. The acquired smoothed data were utilized in independent component analysis (ICA).

**Figure 1:**
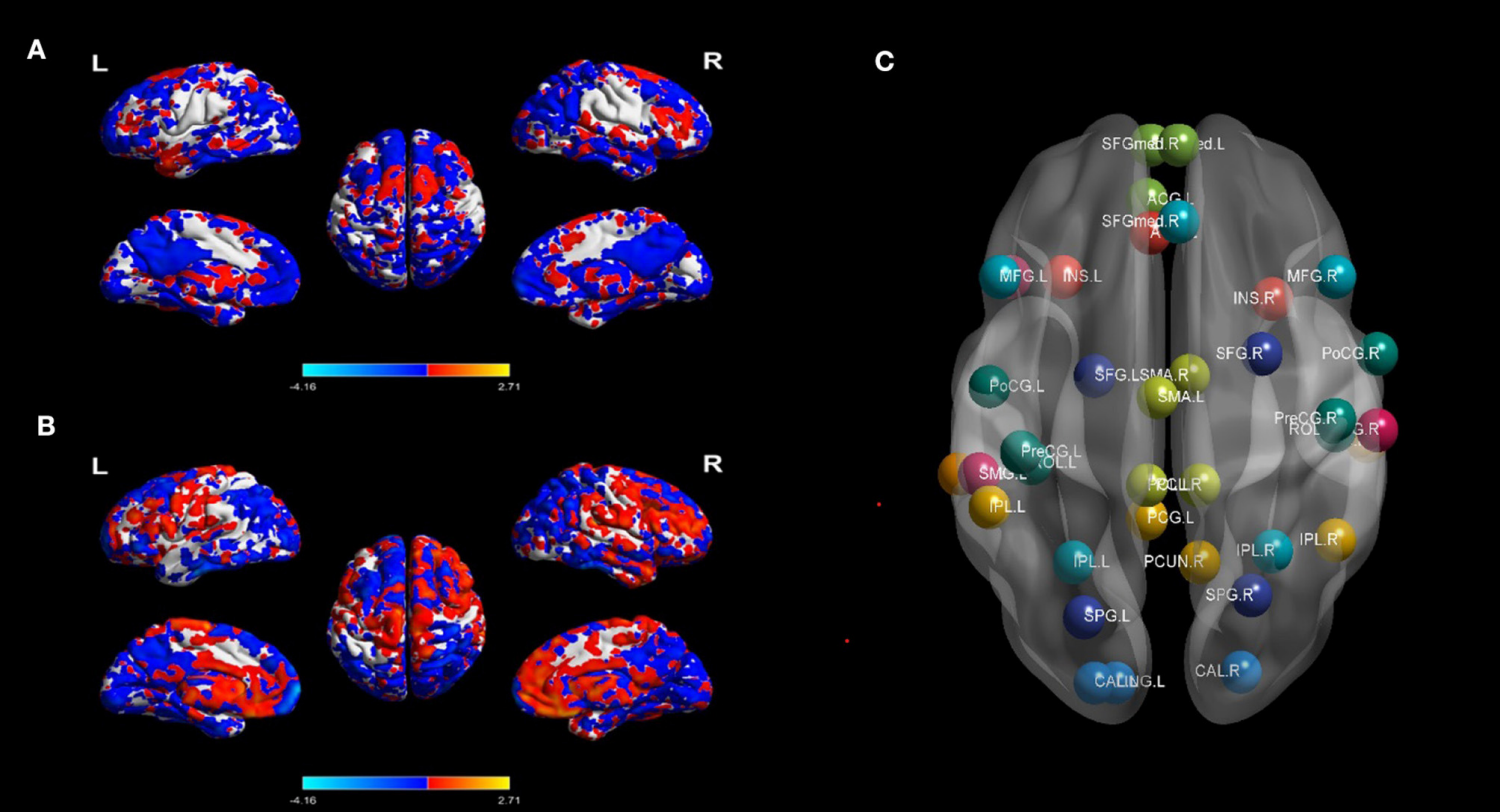
BOLD signal contrasts and seed regions from the 10 RSNs. BOLD signal of HC and MTLE was compared in **(A)**, and BECT-HC contrast in **(B)** (*p* < 0.05, cluster sizes: 50 voxels). Spatial maps of the 10 independent components computed across the entire samples. The color scale represents t values in each spatial component of RSN (maps thresholded at *p* < 0.01, FDR corrected). For networks construction, nodes of interest in the RSNs were extracted according to the peak coordinates of thresholding maps. Different RSNs were depicted as different colors in **(C)**.

The following denoising steps were performed with the unsmoothed images (25): (1) removing the linear trends of time courses; (2) bandpass filtration (0.01–0.08 Hz) to minimize the influence of low-frequency drifts and high-frequency physiological noise; (3) linear regressing out the confounding signals that were unlikely to reflect neural activity, including the head motion effect (26) (Friston 24 parameter), white matter and cerebrospinal fluid signals; and (4) an indispensable “scrubbing” procedure (27). Concretely, in terms of the criteria of framewise displacement (FD) above 0.5 mm, functional imaging data presenting sudden head motion were discarded, together with one volume before and two volumes after the bad volume (28). No patient had fewer than 200 volumes. BOLD signal differences of MTLE-HC and BECT-HC pair-wise contrasts were depicted by REST 18 toolbox (29) to under two simple *t-*test (*p* < 0.05) with cluster sizes as 50 voxels, shown in Figure 1.

#### Cortical Network Identification

Regions that exhibit correlated BOLD fluctuations, i.e., functional connectivity (FC), are regarded as the same functional network (30). According to this theory, we used the group ICA (GICA) method to extract the spatial components of 10 defined resting state networks (RSNs). The Group ICA Of fMRI Toolbox was used for all participants for the group spatial ICA. The data were decomposed into 61 components that were estimated by GIFT, including data reduction by PCA, ICA separation, and back-reconstruction. Two-step PCA was used for data reduction. The maximum likelihood algorithm was used for group-level spatial ICA. A regular algorithm was used for stability analysis, and GICA was used for back-reconstruction. Each subject obtained a spatial component and the corresponding time-series component, and correlation coefficients were converted to a normal distribution by Fishers r-to-z transformation. For each component selection, we obeyed the selection criterion. In particular, ICA selection was independently completed by 2 senior neuroimaging physicians (J.Y. and A.A.) and referred to corresponding templates (31). The spatial maps of each RSN were gathered across all the subjects by the intranetwork connectivity maximum for each cluster of voxels (*p* < 0.01, FDR corrected). The 10 statistic maps were *T*-value connectivity maps. We selected the total 38 ROIs in 10 RSNs based on where was the highest *T*-value in the bilateral sides. The methodology was according to King BR et al. (32). For each local maximum, 38 regions of interest (ROIs) with a 6-mm radius sphere centered on the peak voxel were built with the xjView toolbox (http://www.alivelearn.net/xjview, version 9.6) and REST in MATLAB (Supplementary Figure 1).

#### Subcortical Nucleus Identification

We defined three core subcortical ROIs, the bilateral hippocampi, thalamus and putamen, based on the Harvard-Oxford subcortical atlas in MNI space (33, 34). The subcortial ROIs selection criteria: hippocampus is crucial for MTLE pathological mechanism (35) and putamen and thalamus are all key nuclei for patients with epilepsy. For local motor seizures, epileptogenic networks include thalamocortical circus (36). And putamen is a core nucleus for basal ganglia neuromodulation for motor seizures treatment (37). Therefore, we chose the 3 ROIs in subcortical. Because we did not focus on effects from particular sides of the ROIs, we regarded bilateral ROIs as one seed. In the current study, the hippocampus, putamen and thalamus are abbreviated Hip, Put and Tha, respectively. The selected subcortical ROIs are shown in Supplementary Figure 2. Finally, we obtained 41 spatial mappings of RSNs and 41 average time series of ROIs. The brain networks were visualized with BrainNet Viewer (http://www.nitrc.org/projects/bnv, version 1.6).

#### Intranetwork and Internetwork Analyses

The corresponding time series of the ROI seeds were extracted with REST software, and RSFC in the BECT group, TLE group and HC group was calculated. We obtained three 41 × 41 RSFC matrices and performed Fishers z transformation. For completeness, plots depicting seed-level connectivity (i.e., 41 × 41 matrices) are provided in Supplementary Figure 3. The significance level was set at *p* < 0.05 and corrected for multiple comparisons using network-based statistic (NBS) method (38) (NBS, edge significance: *p* < 0.001, component significance: *p* < 0.05, iterations: 1,000). RSN matrices were acquired by averaging the NBS-corrected FC value (*p* < 0.05) in each group to generate 13 × 13 RSN matrices (32). Cortical internetworks and cortical-subcortical interactions are shown in the off-diagonal line of the 13 × 13 RSN matrices. Intranetwork analysis results were revealed in the diagonal line and indicated the interaction between the inner seeds of each cortical network.

### Statistical Analysis

Demographic information, including age and sex, was compared among the BECT, MTLE and HC groups. Seizure type and duration of epilepsy were compared between BECT and MTLE patients. One-way analysis of variance (ANOVA) was used to test discrepancies in age among the three groups. Chi-square tests were used to compare categorical data, such as sex among the three groups and seizure type distribution between the BECT and TLE groups.

Two-sample *t*-test was used to test the differences of duration between two patient groups. All the above analyses were performed in SPSS 25.0, and *p* < 0.05 was statistically significant.

Ten spatial components of thirty-eight seeds were chosen based on a one-sided one-sample *t*-test (*p* < 0.01, FDR corrected). Correlation maps for each seed in each RSN were computed by correlating regional time series (averaged over all voxels within the seed region) with every voxel in the brain. The 41 time courses, including 3 pairs of subcortical nuclei, were extracted to generate RSFC maps of the 41 × 41 matrix in the three groups. Correlation maps were converted to z maps using Fisher's r-to-z transformation (Supplementary Figure 4). Then, comparisons within each group were performed using one-sided one-sample *t*-tests (*p* < 0.05, NBS corrected) in GRETNA. For detecting intergroups differences, two sample *t*-test was used between MTLE/BECT and HC. And the age differences were taken into account in pairwise comparisons, and the level of significance for group differences was set at *p* < 0.05 (edge *p* < 0.001, NBS corrected).

## Results

### Demographic and Clinical Data

No differences were found among the 3 groups in terms of gender (*p* = 0.951) and seizure type (*p* = 0.056) between the BECT and MTLE groups. For the analysis of duration, BECT shows significant different with MTLE group (*p* < 0.001). One-way ANOVA revealed a significant difference in age among the three groups (*p* < 0.001). A *post hoc* test was performed to find that the BECT-HC contrast (*p* < 0.001) and BECT-MTLE contrast (*p* < 0.001) were significant. The demographic and clinical information of the study participants is presented in Table 1.

### BOLD Signal Contrast and Resting State Networks

The differences of BOLD signal between BECT-HC and MTLE-HC were shown in Figures 1A,B (*p* < 0.05, cluster size: 50 voxels). A total of 61 components were identified by ICA. After selection by visual inspection and templates, 10 valuable components were identified. One-sample *t*-test showed a typical spatial pattern in each RSN and ROIs in each RSN are shown Figure 1C and Table 2. Spatial location of 38 ROIs were detailed in Supplementary Figure 1.

**Table 2:** Resting state functional connectivity networks.

**Brain network and label**	**Abbreviation**	**MNI Coordinates**			***t***
		**X**	**Y**	**Z**	
**Salience network**	**SN**				
Left insula	INS.L	−30	21	−6	16.10
Right insula	INS.R	30	−15	−18	15.66
Anterior cingulum	ACG.L	−5	33	30	15.26
**Post default modal network**	**pDMN**				
Left inferior parietal	IPL.L	−51	−42	42	9.48
Right inferior parietal	IPL.R	48	−51	39	6.45
Post cingulum	PCG.L	−6	−45	30	20.79
Right precuneus	PCUN.R	9	−57	27	23.50
**Anterior default modal network**	**aDMN**				
Anterior cingulum	ACG.L	−6	42	−3	12.16
Left medial prefrontal cortex	SFGmed.L	−5	57	6	16.59
Right medial prefrontal cortex	SFGmed.R	3	57	18	18.32
**Executive control network**	**ECN**				
Left dorsal lateral prefrontal cortex	MFG.L	−48	21	33	16.12
Right dorsal lateral prefrontal cortex	MFG.R	48	21	33	19.03
Medial prefrontal cortex	SFGmed.R	3	36	39	10.63
Left post parietal cortex	IPL.L	−27	−57	39	9.42
Right post parietal cortex	IPL.R	30	−54	45	6.91
**Dorsal attention network**	**DAN**				
Left intraparietal sulcus	SPG.L	−24	−72	51	18.88
Right intraparietal sulcus	SPG.R	24	−66	51	17.73
Left frontal eye field	SFG.L	−21	−6	57	9.04
Right frontal eye field	SFG.R	27	0	57	9.05
**Ventral attention network**	**VAN**				
Left temporoparietal junction	SMG.L	−54	−33	27	11.72
Right temporoparietal junction	SMG.R	60	−21	24	15.53
Ventral frontal cortex	ORBsupmed.L	−45	21	−9	8.27
**Auditory network**	**AN**				
Left superior temporal	STG.L	−60	−33	9	14.81
Right superior temporal	STG.R	57	−24	−3	18.14
**Medial somatomotor network**	**mSMN**				
Left support motor area	SMA.L	−3	−12	63	13.39
Right support motor area	SMA.R	6	−6	48	15.56
Left paracentral lobule	PCL.L	−6	−36	54	16.04
Right paracentral lobule	PCL.R	9	−36	54	15.33
**Lateral somatomotor network**	**lSMN**				
Left precentral gyrus	PreCG.L	−42	−27	51	16.37
Right precentral gyrus	PreCG.R	48	−18	45	17.65
Left postcentral gyrus	ProCG.L	−51	−9	30	15.73
Right postcentral gyrus	ProCG.R	60	0	24	17.87
Left Rolandic operculum	ROL.L	−39	−30	15	21.32
Right Rolandic operculum	ROL.R	48	−21	15	13.32
**Visual network**	**VN**				
Left lingual	LING.L	−15	−90	−9	17.11
Right lingual	LING.R	21	−87	−3	16.46
Left calcarine	CAL.L	−21	−90	−6	15.67
Right calcarine	CAL.R	21	−87	6	14.82

*MNI coordinates and peak connectivity t value for the 38 seed regions extracted from the 10 networks of interest and labels and abbreviations based on the AAL template in MNI space. Ten RSNs were belonged to the following three types of intrinsic functional connectivity pattern: (1) task-positive RSNs (ECN, DAN and VAN); (2) intrinsic RSNs (DMN, SAN); (3) sensory RSNs (SMN, AN and VN). The DMN was subdivided into the anterior DMN (aDMN) and the posterior DMN (pDMN) in our outcomes. And SMN was divided into the medial SMN (mSMN) and the lateral SMN (lSMN)*.

### Functional Connectivity Analysis

#### Internetwork Connectivity

**Figure 2:**
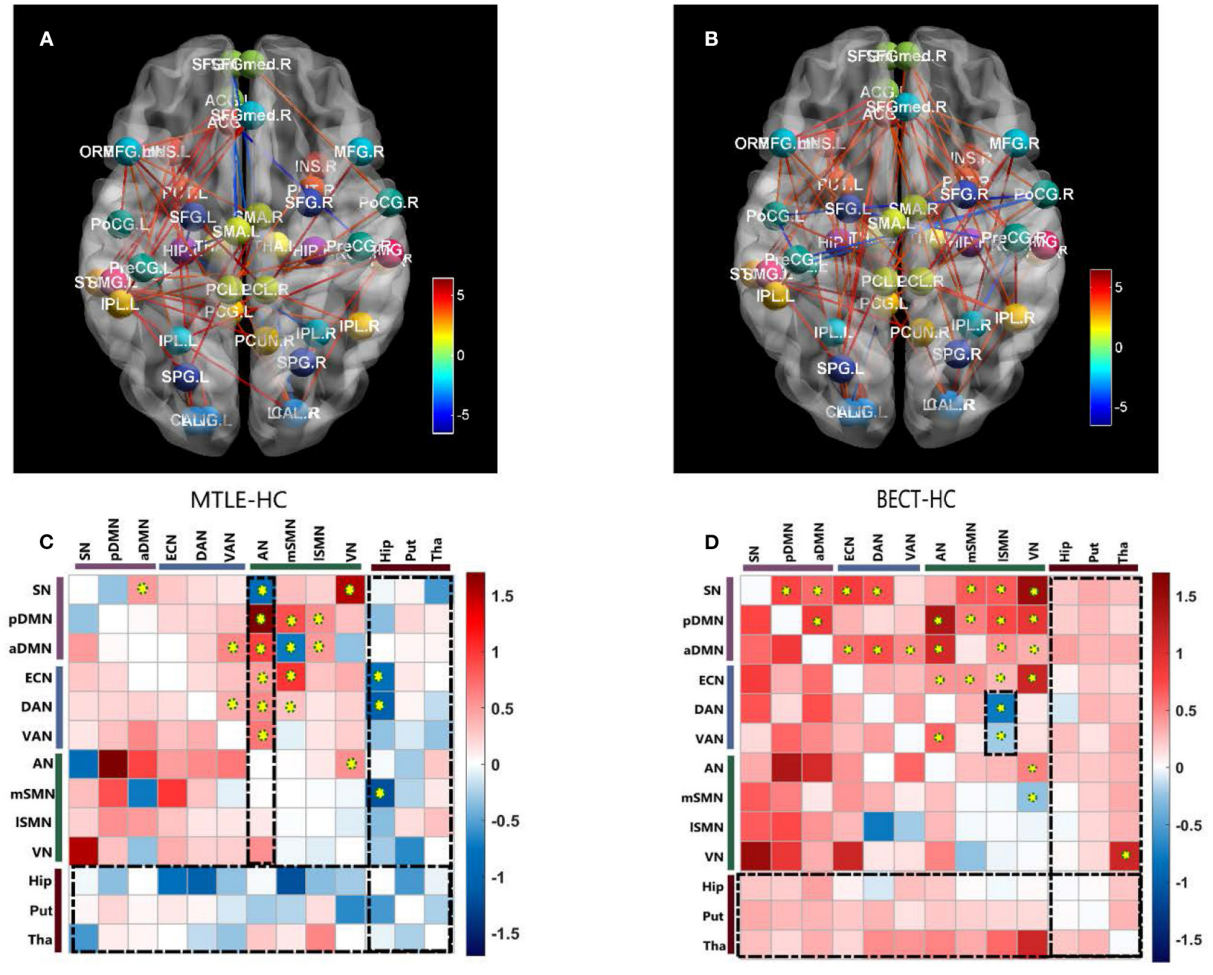
Pairwise comparisons of Internetwork connectivity aberration. Values represented t-statistics between 10 networks and 3 subcortical regions of interest (ROIs: bilateral hippocampus, putamen and thalamus) in the pairwise comparisons of MTLE- HC (**A, C**) and BECT- HC **(B, D)**. Tests of statistical significance were based on two-sample *t*-test corrected for multiple comparisons with a network-based statistic (NBS) threshold set to 0.05. Thredsholded edges and nodes corresponded results of internetwork connectivity **(A, B)**. Specially, we used AAL coordinates to symbolically depict the subcortical ROIs. To better elucidate the general trend, matrix maps were used **(C, D)**. Significant differences are also marked by yellow circuses in the connectivity matrices. The black rectangular box highlighted the characteristic networks, AN in MTLE-HC and lSMN in BECT-HC (details as shown in Figure 3).

Our findings showed different aberrations in network-based interactions in the MTLE (Figure 2A) and BECT (Figure 2B) groups. Compared with the HC group, the MTLE group had lower connectivity between the subcortical hippocampus and task-positive RSNs (ECN and DAN) and sensory RSN (SMN). Notably, the auditory network showed widespread abnormal connectivity with other functional networks, excluding the SMN (Figure 2C), which has been associated with impaired interictal connectivity with the temporal neocortex.

The BECT group showed increased connectivity in the frontoparietal cortex, including the intrinsic RSNs, task-positive RSNs and sensory RSNs (*p* < 0.05, NBS corrected). The SMN showed an increased negative (farther from zero) connectivity with the attention networks (DAN and VAN) and VN (Figure 2D), indicating a relationship to visual attention deficit. Importantly, no particular differences in connectivity were revealed between the cortical networks and the subcortical ROIs, but a trend toward higher interconnectivity was observed (*p* > 0.05, NBS corrected).

#### Intranetwork Connectivity

No differences were found between the MTLE and BECT patients and the HC participants through a two-sample *t*-test. Some critical trends in intranetwork connectivity were revealed in each group. In the MTLE group, intranetwork connectivity was not different from that in the HC group, with the exception of the AN in the lateral temporal lobe. The BECT group showed higher levels of connectivity within most RSNs, such as the SN, pDMN, ECN and DAN (**Figure 4**).

## Discussion

A comparative study of epilepsy compensatory and decompensatory prognosis was conducted in this research. We recruited the patients with BECT and MTLE, the most common types of benign and drug-resistant epilepsies. With the utility of a network-based approach, we demonstrated the different network pattern changes caused by compensation and decompensation, and we also uncovered meaningful networks in a wide range of brain areas with implications for cognitive function.

### RSN Alterations in Patients With MTLE

The current study found that RSNs in the MTLE patients compared with the HCs had lower connectivity with subcortical ROIs, especially the hippocampus, which plays a core role in MTLE. The abnormal connectivity patterns of these networks with the hippocampus were related to functional and structural impairments in the hippocampus. Deactivation compared to the control condition corresponded to decreased synaptic activity, such as that caused by reduced neuronal input from the hippocampus (35). In current study, the condition of impaired consciousness in most patients with MTLE (13/14) might have been caused by subcortical networks with extensive impairments in connectivity with the cortical functional networks (39). These abnormal connections occurred with both task-positive networks and sensory networks. Task-positive networks (e.g., ECN, DAN, and VAN) are dominant in executive control and external attention. Sensory networks are primarily involved in primary somatomotor, somatosensory, visual and auditory processes. Our findings accorded with earlier observations, which showed that MTLE patients demonstrated diffuse neocortical hypometabolism and multitudinous brain connectivity perturbations (40).

**Figure 3:**
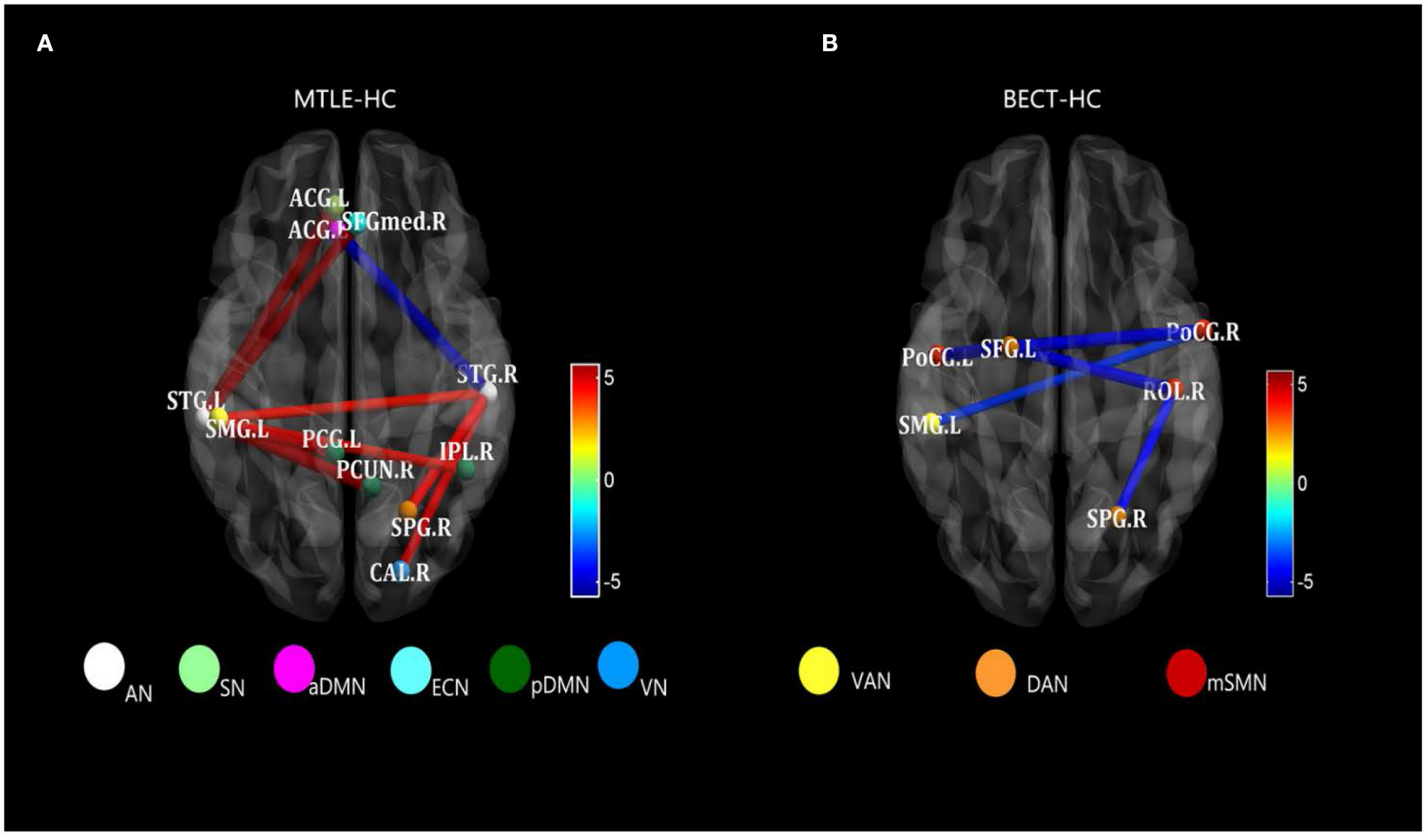
Strongly characteristic networks in MTLE and BECT. We detailed the black rectangular boxes in Figures 2C,D, AN in MTLE-HC and lSMN in BECT-HC. In the analysis of pairwise seed connectivity, bilateral superior temporal gyrus (STG) were found hyperconnectivity with almost functional networks in MTLE-HC **(A)**. Bilateral postcentral gyrus (ProCG.L and ProCG.R) and right Rolandic operculum (ROL.R) showed hypoconnectivity with right intraparietal sulcus (SPG.R within DAN) and left temporoparietal junction (SMG.L within VAN) **(B)**. Tests of statistical significance were based on two-sample *t*-tests (*p* < 0.05, NBS corrected).

The AN showed higher connectivity with other widespread RSNs, including the DMN, which could be explained by the reconfiguration in the lateral temporal area in MTLE patients (41). Blumenfeld and his colleagues used SPECT (39) and found that ictal TLE patients had increased cerebral blood flow (CBF) in the temporal lobe, as well as an increase in CBF in bilateral midline subcortical structures. CBF activity coherence was interpreted as a BOLD signal effect between the lateral temporal and midline areas, manifested as a higher connectivity compared with HCs (see Figure 3A). Thus, damage to lateral temporal lobes, one of the functional network hubs, will affect the sets of functional brain areas at large (42).

### RSN Alterations in Patients With BECT

A resting-state BOLD response was demonstrated to be consistent with interictal seizure discharges in the rolandic region in an EEG-fMRI study (43). Initial studies have shown that the areas of increased connectivity and activity are usually the sensorimotor cortex and immediate regions surrounding the zone. Therefore, a network-based approach may expand our traditional knowledge about the organization of the sensorimotor cortex, especially the interaction between the motor system and the rest of the networks.

The network we refer to as “rolandic” has usually been recognized as a sensorimotor network (SMN) in large-scale RSNs. Effective connectivity studies have suggested that the rolandic area is the key region for the spread of interictal epileptic spikes to distal cortical regions. However, the effect of the rolandic regions is based on the regional distribution of its connectivity among the sets of functional brain areas. Consistent with the research, several studies have found that participants who reported using functional near-infrared spectroscopy (fNIRS) and fMRI also detected a decreased oxyhaemoglobin (HbO) response and an increased deoxyhaemoglobin (HbR) response in the frontal and parieto-occipital lobes, indicating a widespread effect across distributed networks (44, 45).

**Figure 4:**
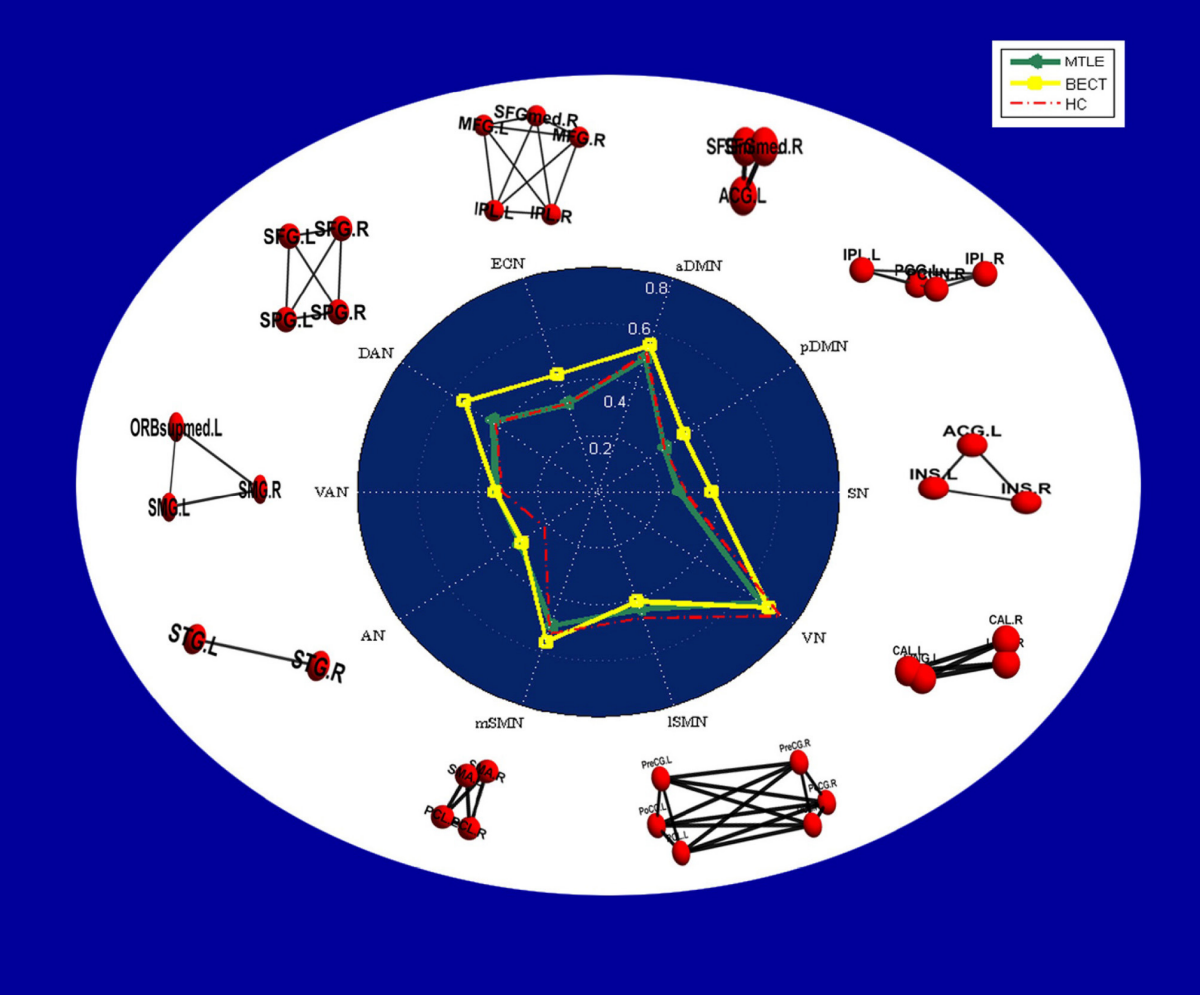
Radar plots showing intranetwork RSFC differences in each group. The values displayed by the dots in the radar plots are the Fisher z-transformed values of Pearson's correlation in each group, and a table of the values is provided in Supplementary Table 1. In the white area, node-edge graphs of 10 RSNs were showed according to Table 2.

Similar findings of discrepant intranetwork connectivity have been previously reported (18, 46), although the current results were not significant compared with the HC group (Figure 4). It was difficult to explain this result, but it might be related to a stronger regional integration (47) in BECT patients. Regardless, there was higher internetwork connectivity among an extensive range of networks, such as the DMN and SAN with other sensory networks (*p* < 0.05, NBS corrected), which corresponded to the loss of cortical global processing (48). In the network-based analysis, excitatory local and global networks indicated that the small-world functional topology was disrupted in BECT patients (49). Notably, no marked lower or higher FC was found between cortical RSNs and subcortical ROIs, which indicated that subcortical core nuclei were not involved in the alterations.

In addition, we uncovered a decreased RSFC between the SMN and attention networks in the hyperconnectivity setting (see Figure 2D) (*p* < 0.05, NBS corrected). The SMN is a motor network as demonstrated in previous studies but is also partially integrated into a multimodal network associated with motor systems and cognitive hubs (50). Impacts on cognition were shown by Caterina et al., who found that BECT patients had impairments in attention (51). Attention control deficits have been related to alterations in the DAN and VAN. A previous study showed increased FC within the VAN in patients when compared with controls (46). This finding was also supported by Jiang et al., who found that children newly diagnosed with BECT showed alterations in brain activity in the attention networks, and the unmedicated group showed increased RSFC in the rolandic network and decreased RSFC in the DAN (52). These findings were in line with attention dysfunction in BECT patients (see Figure 3B). Moreover, the decreased connectivity between the VN and SMN can explain the poor visual spatial memory observed in BECT children (49, 53), which manifests as a loss in integration of the motor network and visual network that forms a multimodal network (54). Moreover, the VN was shown to have a strong correlation with the DMN, indicating that BECT was characterized by possible functional compensatory mechanisms (55) and related to attention-deficit/hyperactivity disorder (ADHD).

Considering the age differences between BECT group (10.42 ± 4.5) and HC group (27.1 ± 4.8), characteristics of RSNs in heathy children and adults were also needed to be discussed as it might influence the results' interpretation to some degree. Resting state studies have shown that children have the same RSNs as adults' and children round 8 years old have strong functional organization, but exhibit immature characteristics (56, 57). Compared with adults, this immature performance was characterized by the functional segregation and the insufficient integration (58, 59). And structure network studies suggested that the approach of network interaction changed from local anatomically regions in children to long-distance cortical interaction in young adults (59, 60). A study of the size of functional networks was found that the number of voxels were more than adults in the majority RSNs and also more widespread (57). In conclusion, it is demonstrated that this kind of segregative pattern in children is less efficient or specialized than adult (61). However, a principle finding in RSNs development was that SMN increased the efficiency of local and global functional connectivity with aging (57, 62). In the current study, BECT patients suffered from the epileptic neural activity in Rolandic area, which could be the reason why healthy adult subjects showed a lower connectivity compared with BECT children. Hence, our primary result was not be interrupted by the age differences.

### Differences in RSNs in Patients With MTLE and BECT

In the internetwork analysis, it was notable that network state differences between the MTLE and BECT patients showed hypoconnectivity between cortical networks and subcortical ROIs in a general setting of lower connectivity in MTLE contrasted against normal cortical-subcortical connectivity and extensive hyperconnectivity among the majority of networks in BECT. This suggested that the two types of epilepsy have completely different brain network patterns that impact clinical outcomes. In our study, patients with MTLE and BECT have totally different severity of clinical manifestations. One of the critical reasons is because the different pathological mechanism. Generally, patients with MTLE have the most common etiology and pathological performance, hippocampus sclerosis, which is irreversible in the course of epilepsy (3). By comparison, BECT is an idiopathic epilepsy without brain structural abnormality and recently research have shown a strong correlation between genetics and BECT development (63). Moreover, different durations of two patient groups were also contributed to different clinical response. Long-term epileptiform discharges would be able to interrupt the brain normal functional activity and also induced structural damage in MTLE (64) while majority of BECT patients remit spontaneously before adolescence.

Connectivity patterns seem to be correlated with the duration and severity of the disease, indicating progressive connectivity reorganization in the context of recurrent seizure activity. BECT was more reflective of a state of increased synchronization in functional network activities, which could be understood as synchronous activity of these related regions that did not stop during the interictal period. Hence, it could conceivably be regarded as a compensatory state of higher synchronization. Moreover, normal cortical-subcortical interactions suggested disruptions confined to cortical functional regions in BECT. In contrast, the MTLE patients showed a widespread state of lower connectivity between RSNs and subcortical ROIs compared with the HCs, which meant a lower global cooperativity that should have relevant functional consequences due to the loss of their normal FC. It could be concluded that MTLE results in more significant disruptions throughout brain networks, and this may help to explain the longer course of the disease, more severe symptoms and worse prognosis of MTLE than BECT through a pathological network mechanism.

We provide new evidence for brain network pattern abnormalities in different epilepsy compensatory states. And we expect that future studies will focus more on the lateral temporal lobes in MTLE and the attention networks in BECT. Furthermore, it seems feasible to use different neuromodulation approaches, for example, transcranial magnetic stimulation (TMS), to investigate these underpinning mechanisms. Michael D. Fox and his colleagues (65) suggested the potential to balance abnormal activity based on RSFC in psychiatric and neurological diseases, including epilepsy (66, 67). Network-based cortical modulation in BECT and MTLE, as typical focal epilepsies, might have the potential to investigate the substrate. Concretely, our findings suggested that the lateral temporal lobes and attention networks are probably valid TMS targets for MTLE and BECT. In addition, transcranial direct current stimulation (tDCS) and other network-based neuromodulation methods, which take these variables into account, will need to be undertaken.

## Limitations

In the current study, our primary focus was on the discrepant state and characteristics of brain functional networks in patients with MTLE and BECT. However, the study findings should be interpreted in the context of their limitations. Firstly, a potential limitation of our study was the small sample size in both epilepsy groups. A larger sample size may produce significant results when the MTLE and BECT groups are compared with the HC group in the intranetwork analysis. Secondly, in our future study, the age discrepancy between BECT and HC needed to be further solved. Thirdly, cognitive state evaluation, such as attention, motion, audition and visual function, is necessary as a Supplementary to verify these dysfunctions. Finally, there is more detailed and related work that could be performed, including effective connectivity and global property analysis in future studies. Future work is required for a full consideration of the above factors.

## Data Availability Statement

The original contributions generated for the study are included in the article/Supplementary Material, further inquiries can be directed to the corresponding authors.

## Author Contributions

JY and CF: conceptualization. CF: methodology and writing—original draft preparation. ZC, AA, and QY: validation. AA and QY: resources. AA and ZC: data curation. JY and WY: writing—review editing, and supervision. WY: funding acquisition. All authors contributed to the article and approved the submitted version.

## Conflict of Interest

The authors declare that the research was conducted in the absence of any commercial or financial relationships that could be construed as a potential conflict of interest.
