# Supplementary material for: Different Functional Network Connectivity Patterns in Epilepsy: A Rest-State fMRI Study on Mesial Temporal Lobe Epilepsy and Benign Epilepsy With Centrotemporal Spike
Source: Front Neurol. 2021 May 28;12:668856. doi: 10.3389/fneur.2021.668856 (PMC8193721; doi:10.3389/fneur.2021.668856)
Supplement: Supplementary file 1 [file Data_Sheet_1.pdf]

Supplementary Material

Different Functional Network Connectivity Patterns in Epilepsy: A Rest-state fMRI Study on Mesial Temporal Lobe Epilepsy and Benign Epilepsy with Centrotemporal Spike

Supplemental Figure 1

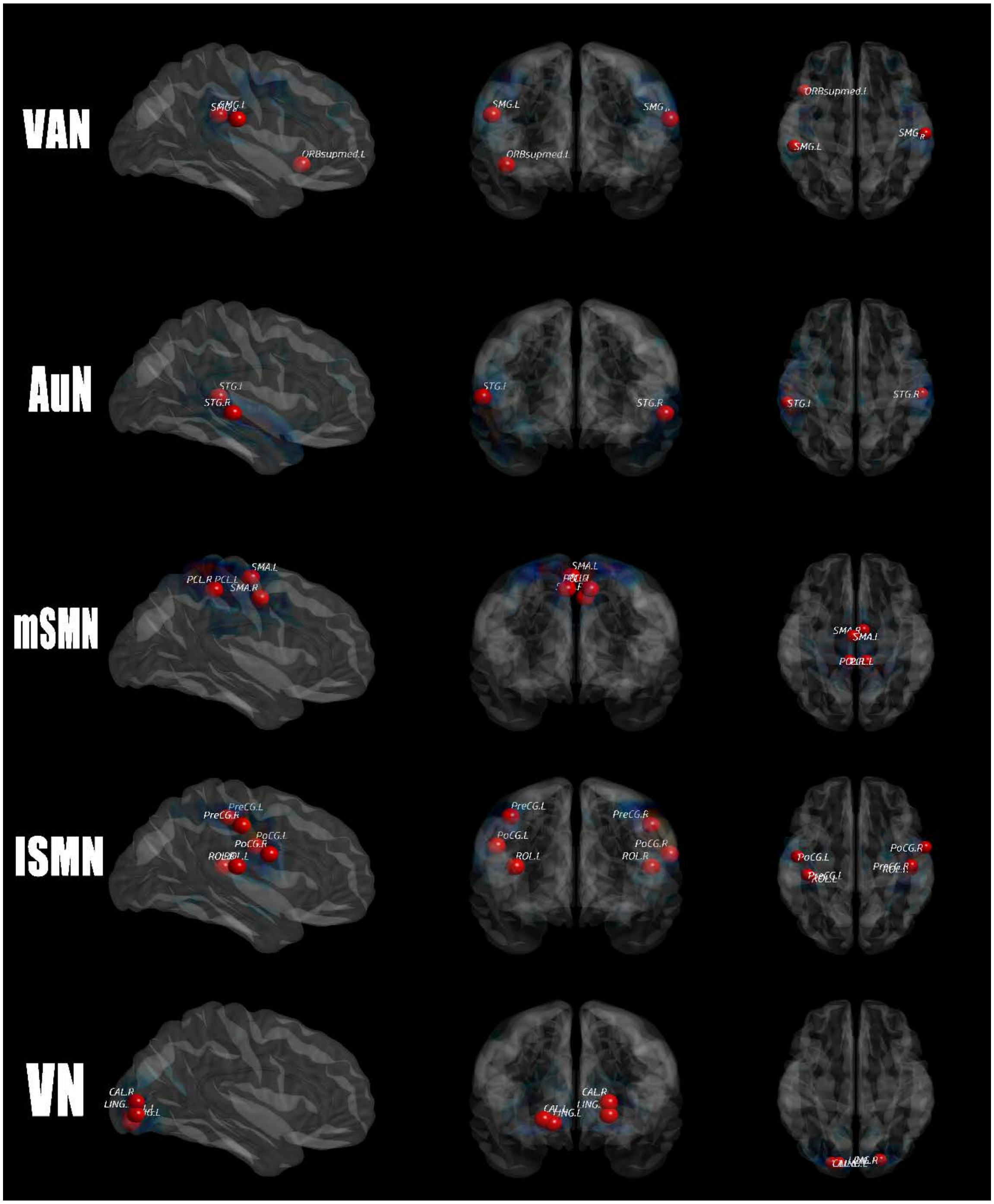

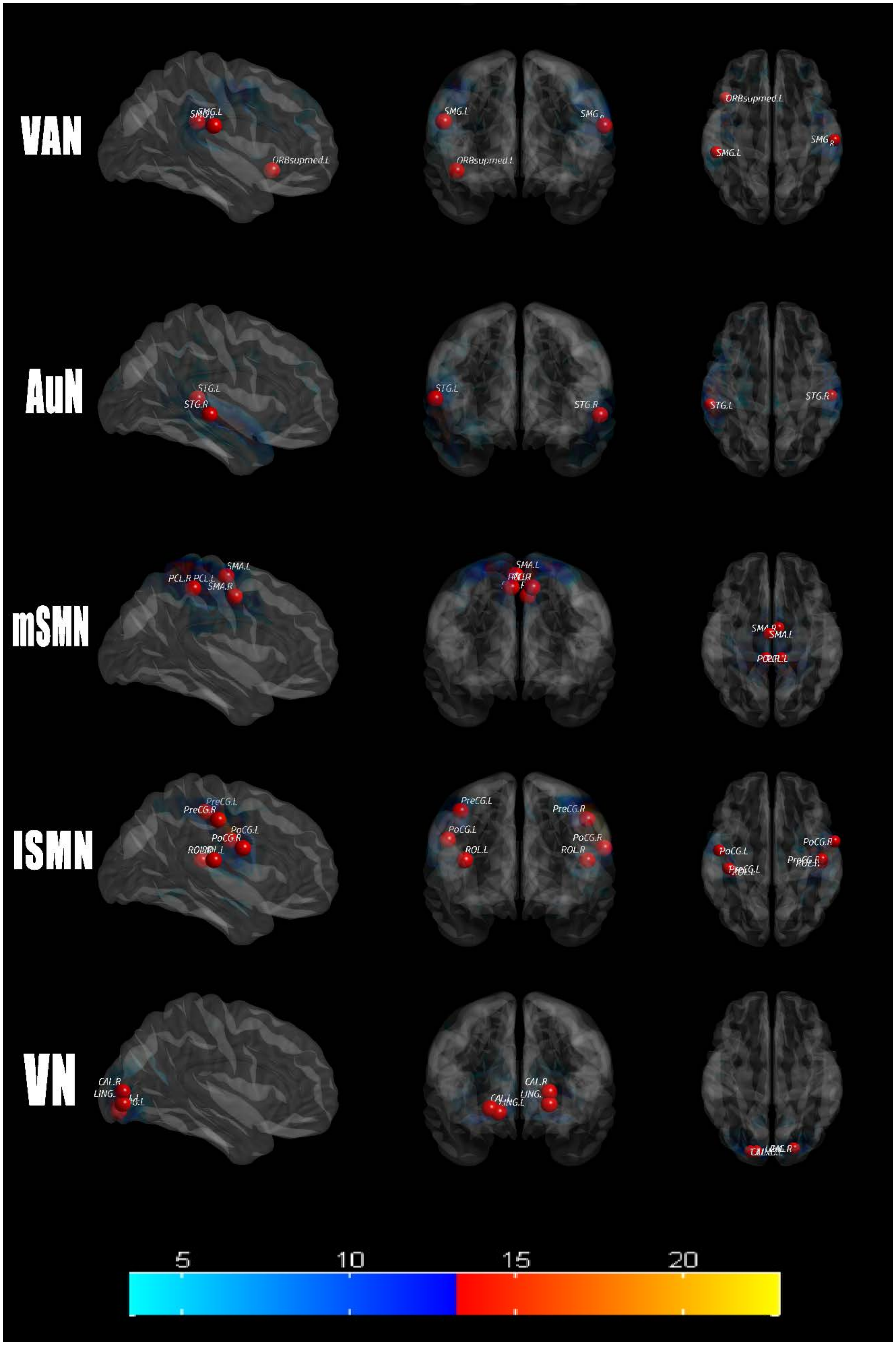
VAN
AuN
mSMN
lSMN
VN
5
10
15
20

After selection by visual inspection and templates, 10 valuable components were identified. 38 regions of interest (ROIs) with a 6-mm radius sphere centred on the peak voxel in each hemisphere of RSNs. One-sample t-test showed a typical spatial pattern in each RSN, and the color bar showed the t-value in each RSN.

Supplemental Figure 2

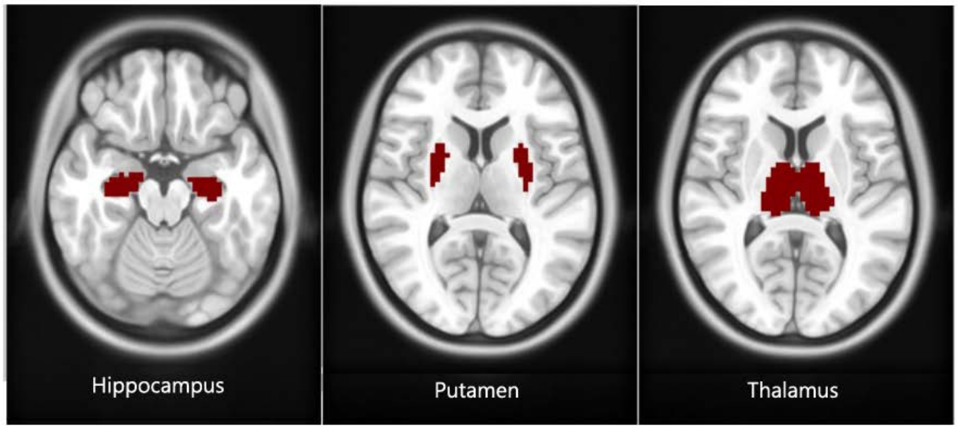

Subcortical regions of interest (ROIs) from Harvard-Oxford subcortical atlas underlaying on coronal plane of the MNI152 template. In the current study, hippocampus, putamen and thalamus abbreviated as Hip, Put and Tha respectively.

Supplemental Figure 3

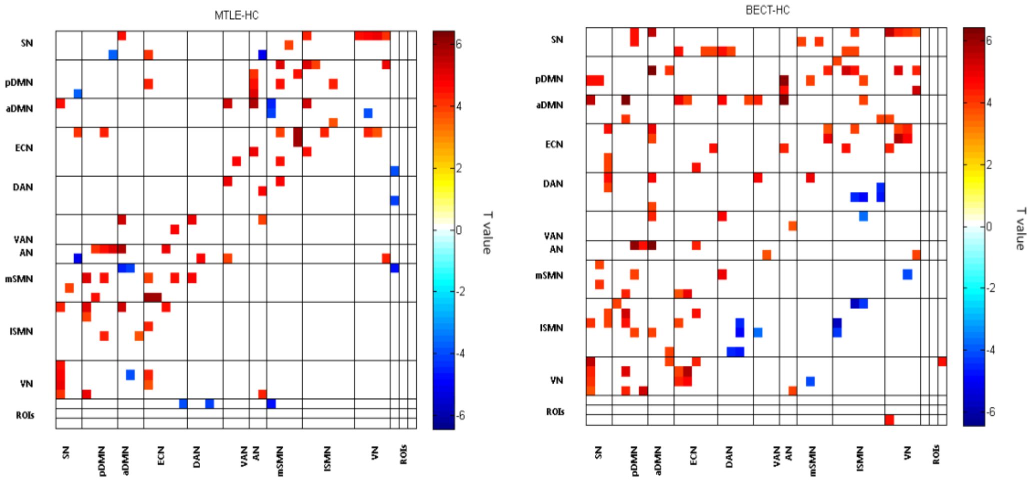

Pairwise comparisons of seeds functional connectivity in MTLE-HC and BECT-HC. Values represent *t*-statistic between the seeds of 10 networks and the 3 subcortical regions of interest (ROIs: bilateral hippocampus, putamen and thalamus). Warmer colors indicate that the disease group exhibited greater connectivity. Tests of statistical significance were based on two-sample *t*-tests corrected for multiple comparisons with a network-based statistic (NBS) threshold set to 0.05.

Supplemental Figure 4

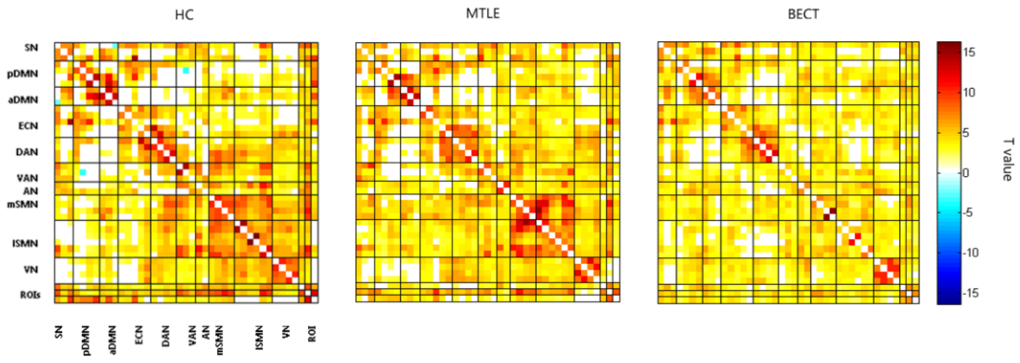

Seed-level resting state connectivity for the 3 groups. Values represent *t*-statistics based on the averaged *z*-transformed correlations between the 41 seeds of interest (see Supplemental Table 1 for specific coordinates on the seeds). Network labels are shown in the bottom left panel and are identical across all subplots. Tests of statistical significance were based on one-sample *t*-tests corrected for multiple comparisons with a network-based statistic (NBS) threshold set to 0.05.

Supplemental Table 1

**Functional networks**	**MTLE**	**BECT**	**HC**
**SN**	**0.27**	**0.38**	**0.29**
**pDMN**	**0.27**	**0.35**	**0.27**
**aDMN**	**0.50**	**0.55**	**0.52**
**ECN**	**0.33**	**0.44**	**0.33**
**DAN**	**0.43**	**0.55**	**0.42**
**VAN**	**0.34**	**0.34**	**0.32**
**AN**	**0.32**	**0.31**	**0.22**

**mSMN**	**0.50**	**0.56**	**0.53**
**lSMN**	**0.44**	**0.41**	**0.47**
**VN**	**0.67**	**0.70**	**0.85**

Pearson’s correlations values by Fisher z transformation within each functional network.
